# Application of PLA-Based Films to Preserve Strawberries’ Bioactive Compounds

**DOI:** 10.3390/foods13121844

**Published:** 2024-06-12

**Authors:** Giuseppina Crescente, Giovanni Cascone, Maria Grazia Volpe, Stefania Moccia

**Affiliations:** 1 National Research Council, Institute of Food Sciences, 83100 Avellino, Italy; giuseppina.crescente@isa.cnr.it (G.C.); giovanni.cascone@isa.cnr.it (G.C.); mgvolpe@isa.cnr.it (M.G.V.); 2 National Agency for New Technologies, Energy and Sustainable Economic Development, 80055 Portici, Italy

**Keywords:** *Fragaria* × *ananassa*, Poly-(Lactic Acid) packaging film, biodegradable packaging, bioactive compounds, shelf-life

## Abstract

Poly-(Lactic Acid) (PLA) is regarded as one of the most promising bio-based polymers due to its biocompatibility, biodegradability, non-toxicity, and processability. The investigation of the potential of PLA films in preserving the quality of strawberries is fully in line with the current directives on the sustainability of food packaging. The study aims to investigate the effects of PLA films on strawberries’ physical and chemical properties, thereby determining whether they can be used as a post-harvest solution to control antioxidant loss, reduce mold growth, and extend the shelf-life of strawberries. Well-designed PLA films with different-sized holes obtained by laser perforation (PLA**_0_**, PLA**_16_** and PLA**_23_**) were tested against a conventional packaging polypropylene (PP) tray for up to 20 days of storage. Weight loss and mold growth were significantly slower in strawberries packed in PLA films. At the same time, PLA-based films effectively preserved the deterioration of vitamin C content, polyphenols and antioxidant activity compared to the control. Furthermore, among all, the micro-perforated PLA film (PLA**_23_**) showed better preservation in the different parameters evaluated. These results could effectively inhibit the deterioration of fruit quality, showing promising expectations as an effective strategy to extend the shelf-life of strawberries.

## 1. Introduction

Strawberries (*Fragaria* × *ananassa*) are one of the most important fruit crops all over the world, widely consumed, both as fresh fruit and as an ingredient in processed products [1,2]. From a nutritional point of view, strawberries are low in calories (32 kcal/100 g), while they represent a good source of minerals, vitamins, and phytochemicals [3,4]. Most phytochemicals are phenolic compounds (flavonoids, anthocyanins, and ellagitannins), and their amount varies greatly based on the cultivar, growth conditions, the degree of ripeness and storage after harvest [5,6]. These bioactive compounds exert a wide range of positive effects, and many studies highlight their health-promoting properties [4,7,8].

Strawberries are very perishable fruits with a short post-harvest life [9]. One of the factors responsible for the perishability of products like fruits or vegetables is that breath and metabolic functions continue even after harvesting [10]. For longer shelf-life, post-harvest treatments that slow down respiration are key [9].

Food packaging plays a key role in preserving the product from deterioration over time, protecting it from microbiological and chemical contamination [11]. Over the past decade, interest in bio-based packaging materials has grown rapidly as a valid alternative to traditional plastic due to their biodegradability, eco-friendliness, non-toxicity, and biocompatibility [12,13]. The bio-based plastic can be either natural or synthetic [13]. Among these latter, Poly-(Lactic Acid) (PLA) is a biodegradable polyester derived from renewable resources, such as corn, potatoes, and sugarcane [14]. It is composed of lactic acid monomers and is generally recognized as safe (GRAS) by the United States Food and Drug Administration (FDA), so it can be used in contact with food [15,16]. 

A significant environmental consideration is that plants absorb carbon dioxide from the atmosphere to produce this polymer, reducing the concentration of greenhouse gases in the environment. When PLA undergoes degradation, it releases the same amount of carbon dioxide into the atmosphere as the plants used to produce it. However, PLA’s carbon dioxide emission rate is very low (1600 kg/metric ton) when compared to polymers produced from petroleum (polypropylene, polystyrene, nylon) [17]. 

In previous studies, PLA has been tested as a packaging material for fresh products. Blueberries stored in PLA bags kept their freshness longer when stored at 10 or 23 °C than those in commercial packages [18]. A study by Zhou et al. (2016) compared the quality changes of fresh-cut melon packaged in PLA and polyethylene terephthalate (PET) at two different temperatures (4 and 10 °C) for 10 days. They found that when the fresh-cut melon was stored at 10 °C for 10 days, PLA containers exhibited greater overall quality preservation than PET ones [19]. The development of PLA hinged-lid trays has proven effective for storing fresh-cut spinach. No differences were found between the two polymeric trays used (PET and PLA) for all parameters evaluated, but the spinach retained its flavor for longer when stored in the PLA ones. Regretfully, the authors reported that condensed water was gathered in the PLA packaging [20]. In strawberries, the effectiveness of PLA active packaging has been evaluated; in particular, PLA films with different percentages of nano-Ag particles effectively safeguarded the freshness of strawberries, with the 5% percentage showing the best results [21]. A PLA composite film with fenugreek essential oil and curcumin was proposed by Subbuvel and Kavan (2022); they observed that the PLA-based composite film exhibited good antibacterial and antioxidant properties [22]. 

The PLA-based films, by themselves, have a high water vapor transmission rate (WVTR) and poor gas permeability [23]. This limitation can be overcome using microporous packaging to control the gas permeability rate and, therefore, CO_2_ and O_2_ levels [24]. In this way, the perforation regulates the breathing of the packaged products, while the transfer of water vapor occurs through the film [23].

This study was conducted in the framework of the European project “*HortiBioPack*” and aims to analyze, for the first time to our knowledge, strawberries’ storage in PLA-based biodegradable packaging for up to 20 days. Here, we tested a new biodegradable film packaging system with macro and micro-laser perforations to prolong the deterioration of strawberries. The use of PLA films with a different number of holes resulted in the preservation of physicochemical properties (weight loss, pH, total titratable acidity, and total soluble solids) and bioactive compounds compared to those in a polypropylene (PP) tray. Differences between various PLA-based perforated films are discussed, highlighting the importance of using an appropriate food packaging system and providing key information for the design of future studies. 

## 2. Materials and Methods

### 2.1. Raw Materials

Strawberries (*Fragaria* × *ananassa* Duch., cv. ‘Candonga’) were harvested at the red ripe stage at “Amendola Carmine” farm (Battipaglia, Salerno, Italy). Fruits were selected to eliminate damaged, shriveled, and unripe fruit. 

### 2.2. Experimental Design

For each experimental group, 100 g of strawberries were weighed and sealed with a heat sealing bar in three different PLA films previously laser-treated in one line along the film to create holes that allowed gas exchange between the packaging and the atmosphere. The PLA films were supplied by the company MACH s.r.l. in Piazzola sul Brenta (Padua, Italy). All three types of films had the same thickness (30 µm) but different numbers and sizes of holes, and therefore a difference in their permeability: (1) PLA**_0_** without holes; (2) PLA**_16_** with 4 macro holes of 6 mm; (3) PLA**_23_** with 15 micro holes of 80 µm. A PP tray was used as a control (CTRL) to simulate conventional packaging used for fruit storage (Figure S1, panels a–g). 

They were placed in a cold room (4 °C) for 20 days, and the analyses were carried out at different times (0, 2, 6, 9, 13, 16 and 20 days) during storage. On each analysis day, two packages of each type of packaging were opened to provide twofold feedback on the results obtained.

### 2.3. Weight Loss

The strawberry weight loss was determined through a technical balance (Europe 500, Gibertini Elettronica; Milan, Italy) during the entire storage period. The percentage weight loss was calculated as follows:

[(W_(*t0*)_ − W_(*t*)_)/W_(*t0*)_] × 100 (1)

where W_(*t0*)_ is the initial sample weight, and W_(*t*)_ is the sample weight at time *t.*

### 2.4. pH, Total Titratable Acidity, and Total Soluble Solids

For all analyses, the strawberries were homogenized with a blender (HR1652/90, Philips; Amsterdam, The Netherlands) without adding water. The pH values of the strawberry homogenate were determined using a digital pH meter (S20 SevenEasy™, Mettler-Toledo; Columbia, MD, USA). 

The total titratable acidity (TTA) was determined according to Win et al. (2021) by diluting the homogenate 100-fold in H_2_O_d_ and then titrating it with a 0.1 M NaOH solution using phenolphthalein as an indicator [25]. Results were expressed as milligrams of citric acid monohydrate equivalents/100 g fresh weight (FW).

The total soluble solids (TSS) expressed as degrees Brix were measured using an Abbe refractometer (RM, Exacta + Optech; Munich, Germany).

### 2.5. Determination of Vitamin C Content

The vitamin C content in strawberries was determined by iodometric titration, as reported by Pagliarulo et al. (2016), with minor modifications [26]. 10 g of strawberry homogenate was mixed with 100 mL of a 6% aqueous oxalic acid solution; the mixture obtained was filtered, and 10 mL of the filtrate was titrated with an iodine 0.01 N solution in the presence of starch paste (1%, *v*/*v*) as an indicator. The results were expressed as mg of ascorbic acid per 100 g FW. 

### 2.6. Extraction and Determination of Total Phenol Content

The extraction was performed following the method reported by Pagliarulo et al. (2016) with slight modifications [26]. Briefly, 25 g of strawberry homogenate was extracted using 50 mL of an acidified solution of MeOH:H_2_O (7:3, *v*/*v*; 1% HCl, *v*/*v*) as the extracting solvent. Two extraction cycles were carried out: the first was performed in an ultrasonic bath (CPX1800H-E, Branson; Danbury, CT, USA) for 15 min; the second, for 16 h at 4 °C under continuous stirring. At the end of each cycle, the sample was centrifuged at 6000 rpm for 15 min (Neya 16R, Remi Elektrotechnik Ltd.; Vasai, India), filtered, and the extraction solvent was stored at 4 °C until further analysis.

The total phenol content (TPC) was determined by the Folin–Ciocalteu method, as reported by Pagliarulo et al. (2016): 20 µL of samples were mixed with 1.58 mL of H_2_O_d_, 0.1 mL of Folin–Ciocalteu reagent and 0.3 mL of 75 g/L of Na_2_CO_3_ [26]. The tubes were mixed and left to react for 30 min in the dark at room temperature. The absorbance was read at 765 nm (DU730 UV-Vis Spectrophotometer, Beckman Coulter; Milan, Italy). Data were expressed as milligrams of gallic acid equivalents (GAEs) per 100 g FW.

### 2.7. Total Anthocyanin Content

The total monomeric anthocyanin content (TAC) was determined following the pH differential method [27]. The extracts were diluted 1:10 with 0.4 M KCl–HCl buffer (pH = 1) and 0.4 M sodium acetate buffer (pH = 4.5). The absorbance of both solutions was measured at 510 nm and 700 nm with a UV/VIS Spectrophotometer (DU 730, Beckman Coulter) after 15 min of incubation in the dark.

The total anthocyanin content was expressed as cyanidin-3-glucoside equivalents per 100 g FW using the molar extinction coefficient and molecular weight of cyanidin-3-glucoside.

(2) $$Anthocyanin\;pigment\left(\frac{\mathrm{mg}}{L}\right)=\frac{A\times MW\times DF\times 10^{3}}{\varepsilon \times l}$$

where *A* = (*A*_520 nm_ − *A*_700 nm_)pH 1.0 − (*A*_520 nm_ − *A*_700 nm_) pH 4.5; *MW* (molecular weight) = 449.2 g/mol for cyanidin-3-glucoside; *DF* = dilution factor established; *l* = pathlength in cm; *ε* = 26,900 molar extinction coefficient, in L × mol^−1^ × cm^−1^, for cyanidin-3-glucoside; and 10^3^ = factor for conversion from g to mg.

### 2.8. Determination of DPPH Radical Scavenging Capacity

The antioxidant capacity was determined by the DPPH^●^ (2,2-Diphenyl-1-picrylhydrazyl) method [28]. In detail, 100 µL of the strawberry extract was mixed with 2.9 mL of DPPH^●^ methanol solution (0.1 mM) and incubated for 20 min in the dark. Subsequently, absorbance was measured at 517 nm (DU 730, Beckman Coulter) in reference to a blank. Trolox was used as a positive standard. The results were expressed in terms of the percentage decrease of the initial DPPH^●^ absorption by the test samples.

### 2.9. Howard Mold Count

Strawberry samples were examined by an optical microscope (Eclipse E200, Nikon; Tokyo, Japan) to detect any mold infection using the Howard mold count method [29]. This method is based on a standardized procedure that counts the percentage of microscopic fields with mold filaments larger than one-sixth of the field diameter [30]. A little section of the sample was transferred to the Howard slide to assess the presence or absence of mold filaments. The slide thus prepared was examined at 100× magnification. The results were expressed as a percentage of mold growth.

### 2.10. Statistical Analysis

The analyses of all experiments were performed in triplicate on each package and expressed as mean ± Standard Error (SE). Graph Pad Prism^®^ 8 software was used for all graphical representations. The data were analyzed using SigmaPlot 15.0 (SPSS Inc., Norman Nie Dale Bent, Hadlai “Tex” Hull, Chicago, IL, USA) software. Statistical differences were evaluated using one-way ANOVA followed by Tukey’s HSD test; a value of *p* < 0.05 was considered significant. 

## 3. Results and Discussion

### 3.1. Weight Loss

Strawberry’s thin skin makes them especially vulnerable to weight loss due to fast dehydration and tissue decomposition [31]. Table 1 displays the weight loss percentage of strawberries stored in different PLA films compared to the control. The evolution of weight loss shows a significant rise during the storage period (20 days) of strawberries kept in a PP tray (control); contrariwise, all PLA films contributed to preventing the transfer of moisture from the fruit (Table 1). At day 20, the highest weight loss was observed for control strawberries (25.81% ± 0.22), while it was the lowest for those in PLA**_23_** (7.65% ± 0.74), followed by PLA**_16_** (8.03% ± 0.25) and PLA**_0_** (11.36% ± 0.63) films, with no significant differences between PLA**_23_** and PLA**_16_**. A significant difference was found between control strawberry samples and PLA films during the storage periods (Table 1). 

There is a direct correlation between weight loss, breathing rate and the rate of moisture evaporation through the fruit skins [32]. Specifically, strawberries have an extremely high respiration rate equal to 25–50 mL CO_2_ kg^−1^ h^−1^ at 10 °C [24,33]. This high respiration rate could be the result of rapid post-harvest deterioration to which these fruits are susceptible [9]. The laser macro and micro-perforated PLA reduces the transfer of humidity by creating a barrier layer on the surface of the fruit and is, therefore, particularly advantageous for a lower weight loss of the fruit during its storage.

### 3.2. pH, Total Titratable Acidity, and Total Soluble Solids

Table 2 shows the pH, TTA and TSS of strawberries during storage in different packages. The pH of strawberries in all packaging ranges from 3.32 ± 0.01 to 3.77 ± 0.00, and these values are on average with those of ripe strawberries (Table 2, panel a) [34]. The recorded values, on average, are consistent with the work results of Musto and Satriano (2010), who reported a pH value of 3.5 for strawberries cv. ‘Candonga’ [35].

Little variations occur in the TTA value of control and PLA-packed strawberries during the storage period. The percentages of TTA range from 0.35% ± 0.00 to 0.95% ± 0.00 (Table 2, panel b). The acidity of strawberries is strongly related to the concentration of organic acids, such as malic acid and citric acid [36]. These values fall within the range reported in a previous work by Green (1971) (from 0.6 to 2.3%) and are close to the values found by Cordenunsi et al. (2003) (between 0.6 and 0.7) and Mirahmadi et al. (2011) (between 0.52 and 0.86) [37,38,39]. Except for strawberries kept in PLA**_0_**, all samples showed higher citric acid amounts than the initial period at the end of storage time. This trend was also highlighted by Nunes et al. (2006) [34]. Furthermore, in most cases, strawberries packaged in PLA films had a significantly lower level of citric acid than control ones. However, there is clear evidence that cultivar affects pH and citric acid content during storage [34,38,39].

The TSS content depends on the ripeness of the fruits [40]. The obtained results show that over time, the TSS increases slightly and then decreases (Table 2, panel c). For some of them, different PLA films appear to statistically influence this parameter compared to the control.

### 3.3. Vitamin C Content

Strawberries are an important source of vitamin C for human nutrition [41]. However, the ease with which it degrades is an important factor affecting the nutritional quality and shelf-life of strawberry juice [42]. Post-harvest oxidation of vitamin C in plant tissues has been shown to depend on several factors, such as temperature, packaging, and storage time [43].

**Figure 1:**
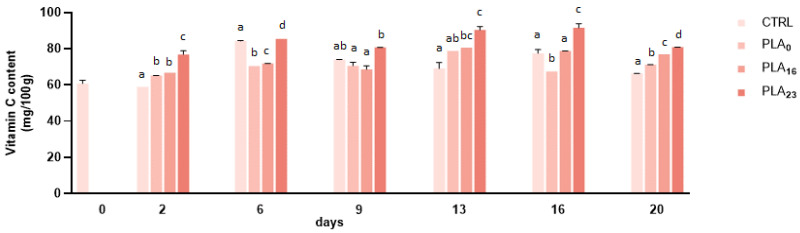
Vitamin C content (mg/100 g FW) of strawberries stored in a PP tray (CTRL), PLA**_0_**, PLA**_16_**, and PLA**_23_** films for 20 days. Data are expressed as mean ± SE. Different letters indicate significant differences (*p* < 0.05). The absence of letters indicates non-significant differences between samples.

The content of vitamin C in strawberries cv. ‘Candonga’ ranges from 53.00 ± 0.01 to 90.51 ± 1.40 mg/100 g FW during the storage period (Figure 1). The experimental data are comparable to the data reported in the literature; in fact, the content of vitamin C was similar to those found by Cervantes et al. (2020) (average 59.65 ± 3.91) for strawberries cv. ‘Candonga’, even if there is variability among the different cultivars [34,38,44].

In our study, during storage, the vitamin C content seems to be influenced by the different packaging employed: in control strawberries, it decreases starting from the ninth day, while in strawberries in PLA films, it increases up to 13 days (with the exception for a slight decline on day 9) and decreased starting from 16 days (Figure 1). At the end of storage, the vitamin C level was better preserved in all types of PLA films compared to the control, and PLA**_23_** was able to prevent the loss of vitamin C at all storage times considered. Furthermore, the increase recorded for PLA**_23_** was statistically significant compared to the other types of PLA films. 

The loss of vitamin C is connected to the dehydration of the fruit [45]: the experimental data showed that strawberries in PP containers (weight loss of 26%) have a higher loss of vitamin C. 

### 3.4. Effect of Different Packaging on TPC and TAC 

**Figure 2:**
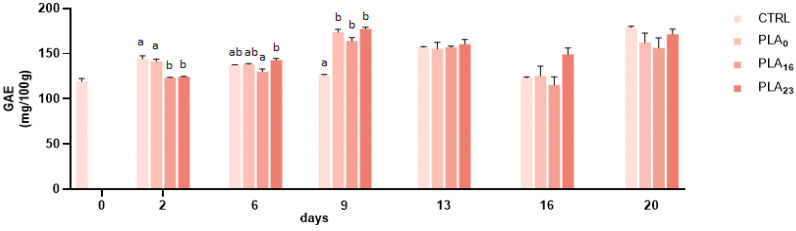
TPC of strawberries stored in a PP tray (CTRL), PLA**_0_**, PLA**_16_**, and PLA**_23_** films for 20 days. Data are expressed as mean ± SE. Different letters indicate significant differences (*p* < 0.05). The absence of letters indicates non-significant differences between samples.

The TPC, evaluated by the Folin–Ciocalteu test, ranged between 119.26 ± 2.26 to 186.61 ± 6.23 mg GAE/100 g FW. The data obtained show that the phenolic content increases during storage in all packages used (Figure 2). Throughout storage, strawberries’ total phenolic content can be preserved or modified, depending on the temperature and storage time [46]. The observed rise is in line with the findings of Koyama et al. (2022), who monitored the TPC of strawberries in various conditions, including at 0 °C for 10 days [47]. Previous work by Ayala-Zavala et al. (2004) reported an ongoing increase in total phenols when strawberries were stored at 10 °C and 5 °C for 13 days after harvesting [48].

Furthermore, the characteristics of the packages employed have an impact on the O_2_ level, with different consequences on TPC until the end of the storage; in fact, when strawberries are exposed to high levels of oxygen, phenolic compounds may accumulate, but may also oxidize after prolonged treatment [49]. Accordingly, strawberries packaged in PLA**_0_** showed the lowest increase (1.15-fold); on the other hand, PLA**_23_** was the highest (1.38-fold). The different result could be due to the presence/absence and size of the perforation which allows the exchange of gases, thus guaranteeing the natural transpiration of the product.

The effect of oxygen on strawberry phenols was evaluated by Zheng et al. (2007), who highlighted that oxygen concentrations above 60 kPa result in an increase in total phenolics during the first 7 days of storage [49].

**Figure 3:**
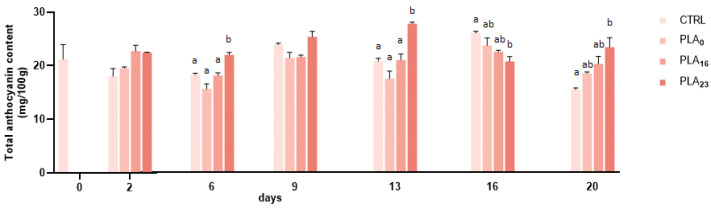
Total anthocyanin content (mg/100 g FW) of strawberries stored in a PP tray (CTRL), PLA**_0_**, PLA**_16_**, and PLA**_23_** films for 20 days. Data are expressed as mean ± SE. Different letters indicate significant differences (*p* < 0.05). The absence of letters indicates non-significant differences between samples.

The effect of different packaging on the TAC of strawberries during storage is shown in Figure 3. The TAC found agrees with the literature data and ranges between 15.40 ± 0.20 and 29.27 ± 0.45 [49,50]. The trend during storage was irregular for all packages used. This fluctuation was also found by Bal and Ürün (2021) in strawberries differently coated throughout the 15 storage days [51].

The very variable trend of anthocyanins over time is consistent with the results obtained by Ayala-Zavala et al. (2004), where temperatures between 0 and 6 °C led to anthocyanin levels that vary over time and are lower than storage at 10 °C [48].

### 3.5. Antioxidant Activity

**Figure 4:**
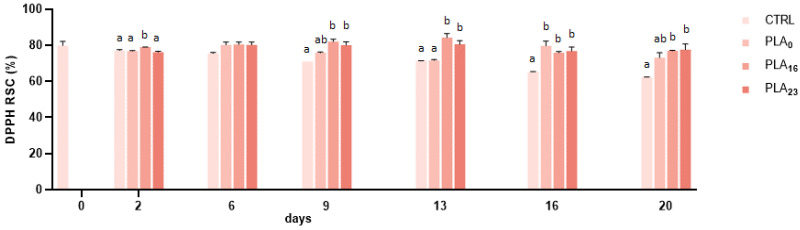
Radical Scavenger Capacity (RSC, %) of strawberries stored in a PP tray (CTRL), PLA**_0_**, PLA**_16_**, and PLA**_23_** films for 20 days. Data are expressed as mean ± SE. Different letters indicate significant differences (*p* < 0.05). The absence of letters indicates non-significant differences between samples.

The antioxidant activity of strawberries stored in different packages was assessed by the DPPH method, and the results are shown in Figure 4. The inhibition percentage decreased during the storage period, especially for strawberries packed in a PP tray (CTRL), by 21.63% compared to the starting point; on the contrary, it was better preserved in PLA packaging with a reduction of 2.18% in PLA**_16_** and an increase of 2.20% in PLA**_23_** compared to the starting point (Figure 4). 

Strawberries are rich in bioactive molecules especially flavonoids, tannins, and phenolic acids with antioxidant activity [8,52]. In addition to these molecules, vitamin C, a non-phenolic molecule, accounts for 30% of strawberries’ antioxidant activity [53]. The antioxidant activity is the result of the content of phenols and anthocyanin, as well as total vitamin C [8]. 

### 3.6. Effect of Different Packaging on Molds

Strawberries are perishable fruits, susceptible to spoilage by *Botrytis cinerea* after harvest, which limits their commercialization and consumption [54]. It is a pathogenic fungus that causes gray molds in several plants [55]. To reduce deterioration, fruit can be given protective coatings, stored under controlled conditions, or properly packaged [18]. 

**Figure 5:**
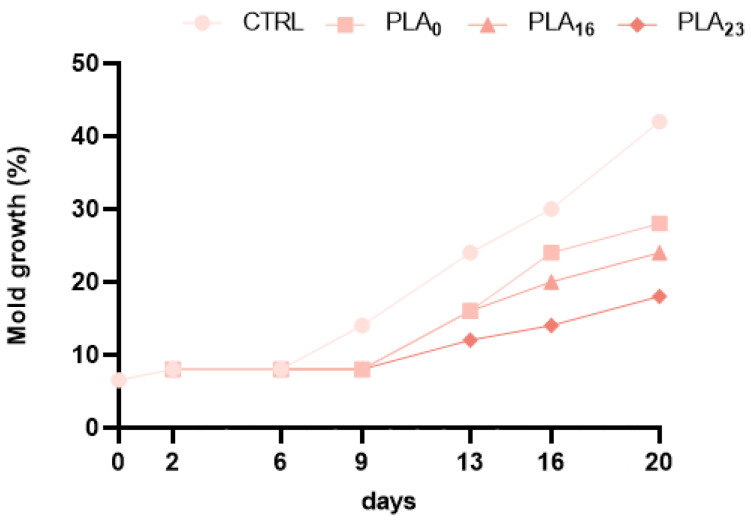
Mold growth percentage of strawberries during 20 days of storage in different packaging. Symbol legend: (●) CTRL, (■) PLA**_0_**, (▲) PLA**_16_**, and (♦) PLA**_23_** films. Data are expressed as mean ± SE.

The results obtained show that for the control, a significant development of mold occurred during storage (from 7.50% at t2 to 41.50% at t20). Mold growth was statistically significantly slowed by PLA films, especially PLA**_23_** (from 8.00% at t2 to 18.50% at t20) (Figure 5; Table S1). Furthermore, the variations between the different PLA film types became significant, starting at 16 days, with PLA**_23_** slowing mold growth better.

Previous work had highlighted the ability of PLA packaging to counteract the growth of yeasts and molds in freshly cut red cabbage. The PLA’s boosting bactericidal activity could be explained by its higher polyphenol oxidase (PPO) activity, which promotes the conversion of phenolic substances into bactericidal quinones [56]. 

## 4. Conclusions

Strawberries are one of the most difficult fruits to preserve because, once ripe and picked from the plant, they must be consumed or processed fast. Even if refrigerated, they retain their characteristic scent for a short time, become darker in color due to pigment oxidation, and the pulp gradually softens. 

Our approach involves the use of sustainable food packaging, and the evaluation of how different perforated PLA films influence the degradation process of the fruit. This strategy is linked to the theory of implementing sustainable food packaging as the European Union’s objective by 2030, based on the concept that the world of industry and companies must prepare for the ecological transition by switching to environmentally friendly packaging, which must also protect the nutraceutical properties of horticultural crops such as strawberries.

Based on the results obtained, PLA films are able to prevent fruit weight loss during storage. This outcome not only can have an impact on the quality of the product, but also represents a significant economic aspect for farms, which seems promising from the point of view of food waste and nutritional value. At the end of the storage period, the strawberries in PLA films, especially in the micro-perforated PLA film (PLA_23_), were preserved better than in the PP tray. In fact, strawberries packaged in PLA films showed reduced fungal growth, and, at the same time, the content of vitamin C, polyphenols and antioxidant activity was preserved for up to 20 days of storage. 

## Figures and Tables

**Table 1:** Weight loss percentage of strawberries during 20 days of storage at 4 °C in different packaging (CTRL, PLA**_0_**, PLA**_16_**, and PLA**_23_** films).

Weight Loss (%)				
Days	CTRL	PLA_0_	PLA_16_	PLA_23_
2	2.57 ± 0.25 ^a^	0.63 ± 0.03 ^b^	0.61 ± 0.01 ^b^	0.60 ± 0.04 ^b^
6	8.54 ± 0.03 ^a^	3.73 ± 0.11 ^b^	2.70 ± 0.05 ^c^	2.69 ± 0.18 ^c^
9	13.43 ± 0.04 ^a^	6.07 ± 0.21 ^b^	4.31 ± 0.07 ^c^	4.18 ± 0.29 ^c^
13	17.98 ± 0.05 ^a^	8.39 ± 0.32	5.39 ± 0.19 ^c^	5.49 ± 0.38 ^c^
16	20.42 ± 0.03 ^a^	9.46 ± 0.36 ^b^	6.23 ± 0.19 ^c^	6.17 ± 0.42 ^c^
20	25.81 ± 0.16 ^a^	11.36 ± 0.45 ^b^	8.03 ± 0.18 ^c^	7.65 ± 0.52 ^c^

Data are expressed as mean ± SE. Within each row, overall means with different superscript letters are significantly different (*p* < 0.05).

**Table 2:** pH, TTA and TSS of strawberries stored in different packaging (CTRL, PLA**_0_**, PLA**_16_**, and PLA**_23_** films).

**(a)**				
**pH**				
**Days**	**CTRL**	**PLA_0_**	**PLA_16_**	**PLA_23_**
0	3.50 ± 0.01			
2	3.38 ± 0.01 ^a^	3.49 ± 0.01 ^b^	3.63 ± 0.01 ^c^	3.61 ± 0.01 ^c^
6	3.52 ± 0.01 ^a^	3.62 ± 0.01 ^b^	3.42 ± 0.01 ^c^	3.49 ± 0.01 ^ac^
9	3.47 ± 0.00 ^ab^	3.44 ± 0.01 ^a^	3.51 ± 0.01 ^bc^	3.57 ± 0.01 ^c^
13	3.32 ± 0.01 ^a^	3.52 ± 0.00 ^b^	3.73 ± 0.01 ^c^	3.54 ± 0.00 ^b^
16	3.60 ± 0.01 ^a^	3.53 ± 0.01 ^b^	3.50 ± 0.01 ^b^	3.62 ± 0.01 ^a^
20	3.46 ± 0.01 ^a^	3.77 ± 0.00 ^b^	3.51 ± 0.00 ^a^	3.57 ± 0.01 ^c^
**(b)**				
		**T** **TA**		
**Days**	**CTRL**	**PLA_0_**	**PLA_16_**	**PLA_23_**
0	0.35 ± 0.00			
2	0.81 ± 0.00 ^a^	0.67 ± 0.00 ^b^	0.43 ± 0.02 ^c^	0.56 ± 0.01 ^b^
6	0.81 ± 0.00 ^a^	0.63 ± 0.01 ^b^	0.47 ± 0.00 ^c^	0.84 ± 0.02 ^a^
9	0.63 ± 0.02	0.78 ± 0.00	0.65 ± 0.00	0.68 ± 0.04
13	0.95 ± 0.00 ^a^	0.69 ± 0.01 ^b^	0.47 ± 0.00 ^c^	0.42 ± 0.00 ^d^
16	0.79 ± 0.02 ^a^	0.61 ± 0.01 ^b^	0.76 ± 0.01 ^a^	0.59 ± 0.00 ^b^
20	0.88 ± 0.02 ^a^	0.62 ± 0.01 ^b^	0.70 ± 0.01 ^b^	0.69 ± 0.00 ^b^
**(c)**				
		**TSS**		
**Days**	**CTRL**	**PLA_0_**	**PLA_16_**	**PLA_23_**
0	8.63 ± 0.09			
2	10.50 ± 0.00 ^a^	9.38 ± 0.09 ^b^	9.38 ± 0.09 ^b^	8.38 ± 0.09 ^c^
6	10.25 ± 0.25 ^a^	8.25 ± 0.25 ^b^	10.75 ± 0.18 ^a^	10.60 ± 0.07 ^a^
9	11.63 ± 0.09 ^a^	8.50 ± 0.00 ^b^	10.13 ± 0.37 ^ab^	9.62 ± 0.13 ^b^
13	8.75 ± 0.00 ^a^	9.13 ± 0.09 ^a^	10.13 ± 0.09 ^b^	10.61 ± 0.08 ^b^
16	8.50 ± 0.00 ^a^	9.25 ± 0.00 ^b^	8.63 ± 0.09 ^a^	8.59 ± 0.06 ^a^
20	8.38 ± 0.09 ^a^	7.13 ± 0.09 ^bc^	7.25 ± 0.00 ^b^	6.60 ± 0.07 ^c^

Data are expressed as mean ± SE. Within each row, overall means with different superscript letters are significantly different (*p* < 0.05). The absence of letters indicates non-significant differences between samples.

## Data Availability

The original contributions presented in the study are included in the article/Supplementary Materials, further inquiries can be directed to the corresponding author.

## Supplementary Materials

The following supporting information can be downloaded at https://www.mdpi.com/article/10.3390/foods13121844/s1, Figure S1: Strawberry fruits (*Fragaria* × *ananassa* Duch., cv. ‘Candonga’) in different packages: CTRL, PLA**_0_**, PLA**_16_**, and PLA**_23_** films (panel a: day 0; panel b: day 2; panel c: day 6; panel d: day 9; panel e: day 13; panel f: day 16; panel g: day 20); Table S1: Mold growth percentage of strawberries during 20 days of storage at 4 °C in different packaging (CTRL, PLA**_0_**, PLA**_16_**, and PLA**_23_** films).
