# Supplementary material for: Application of PLA-Based Films to Preserve Strawberries’ Bioactive Compounds
Source: Foods. 2024 Jun 12;13(12):1844. doi: 10.3390/foods13121844 (PMC11202936; doi:10.3390/foods13121844)
Supplement: Supplementary file 1 [file foods-13-01844-s001.zip › foods-3038266-supplementary.pdf]

Supplementary materials

a)

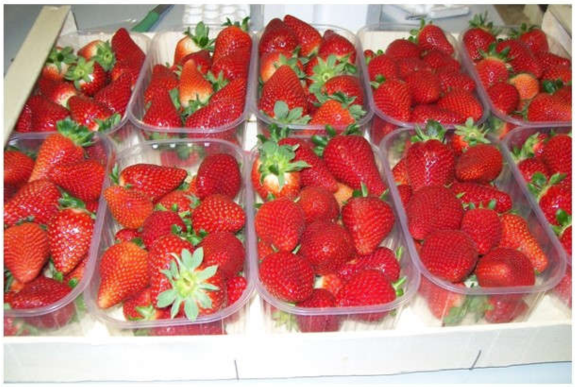

b)

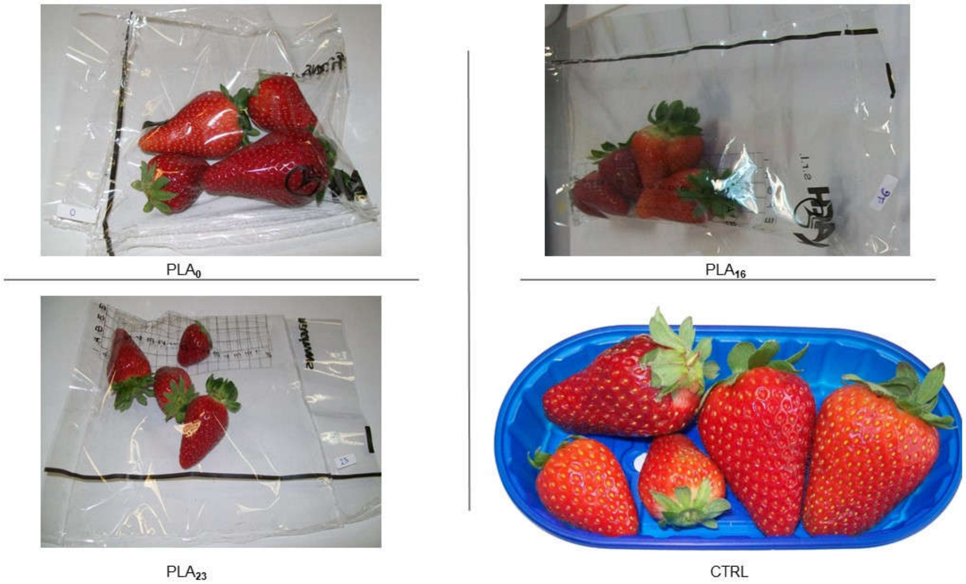

c)

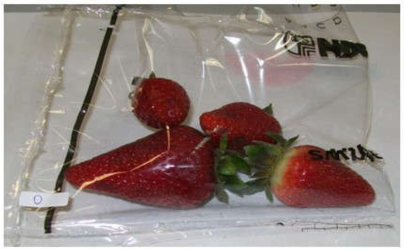

PLA_0

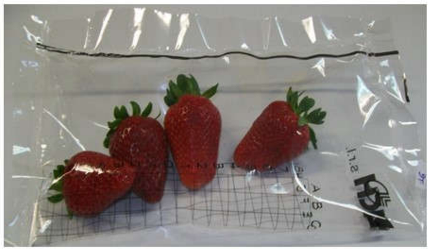

PLA_{16}

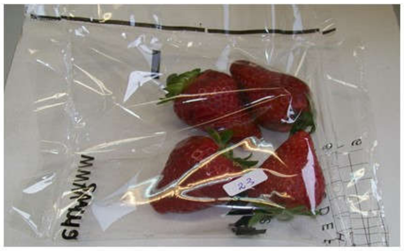

PLA_{23}

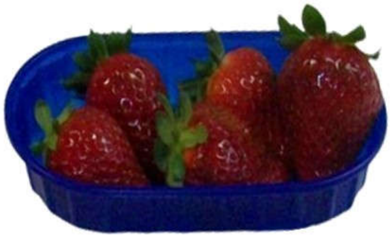

CTRL

d)

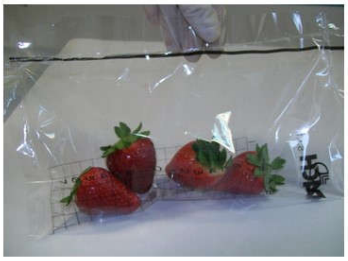

PLA_0

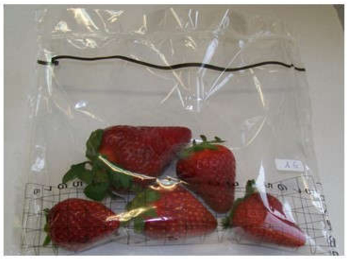

PLA_{16}

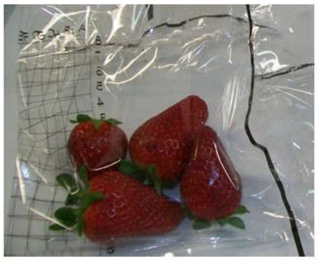

PLA_{23}

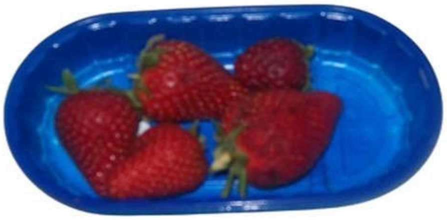

CTRL

e)

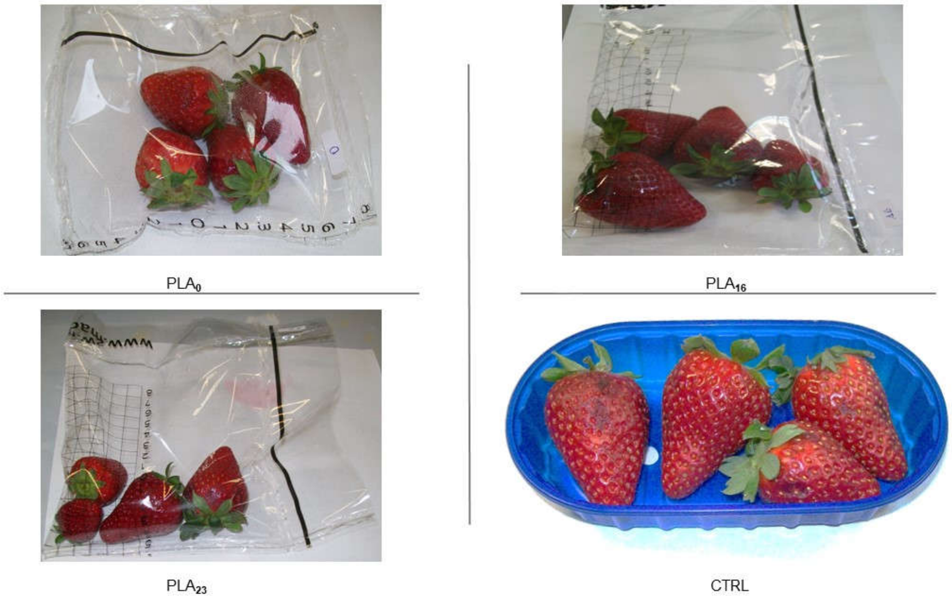

f)

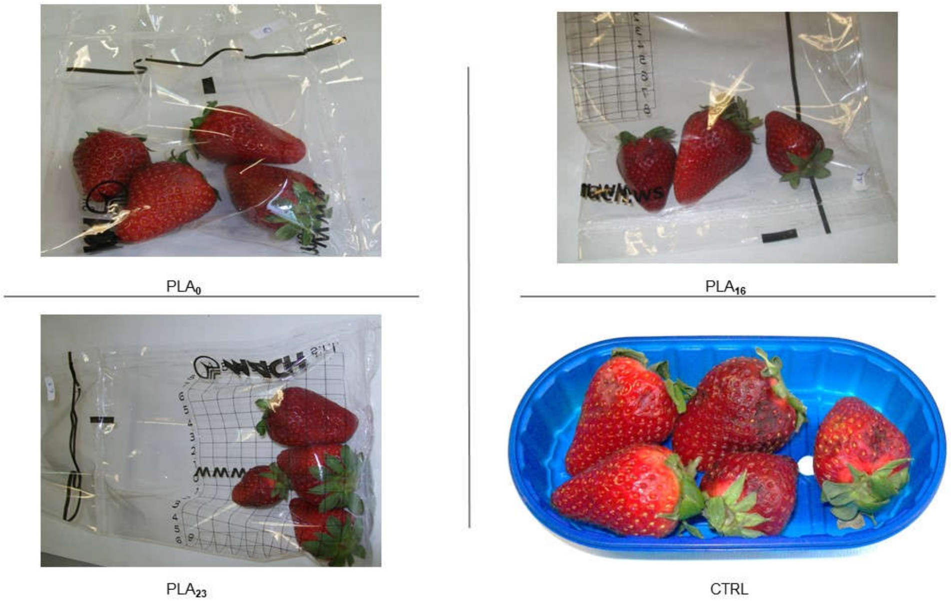

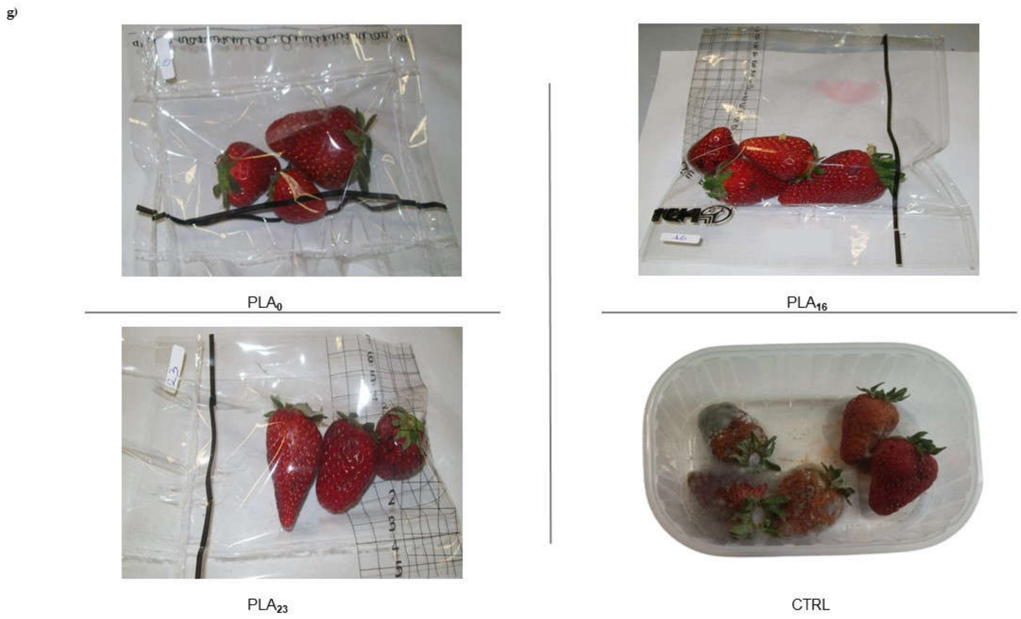

Figure S1. Strawberry fruits (*Fragaria* × *ananassa* Duch., cv. 'Candonga') in different packages: CTRL, PLA_0, PLA_{16}, and PLA_{23} films (panel a: day 0; panel b: day 2; panel c: day 6; panel d: day 9; panel e: day 13; panel f: day 16; panel g: day 20).

Table S1. Mold growth percentage of strawberries during 20 days of storage at 4 °C in different packaging (CTRL, PLA_0, PLA_{16}, and PLA_{23} films).

		Mold growth (%)		
**Days**	**CTRL**	**PLA_0**	**PLA_{16}**	**PLA_{23}**
0	6.50 ± 0.40			
2	7.50 ± 0.35	8.00 ± 0.10	8.00 ± 0.00	8.00 ± 0.00
6	7.50 ± 0.35	8.00 ± 0.15	8.00 ± 0.07	8.00 ± 0.26
9	13.50 ± 0.35^a	8.00 ± 0.30^b	8.00 ± 0.09^b	8.00 ± 0.40^b
13	23.00 ± 0.71^a	15.50 ± 0.35^b	15.00 ± 0.10^b	11.50 ± 0.35^b
16	29.50 ± 0.35^a	24.00 ± 0.00^b	20.50 ± 0.50^c	13.50 ± 0.50^d
20	41.50 ± 0.35^a	27.50 ± 0.50^b	24.00 ± 0.00^c	18.50 ± 0.50^d

Data are expressed as mean ± SE. Within each row, overall means with different superscript letters are significantly different ($p < 0.05$). The absence of letters indicates non-significant differences between samples.
